# The deduced role of a chitinase containing two nonsynergistic catalytic domains

**DOI:** 10.1107/S2059798317018289

**Published:** 2018-01-01

**Authors:** Tian Liu, Weixing Zhu, Jing Wang, Yong Zhou, Yanwei Duan, Mingbo Qu, Qing Yang

**Affiliations:** a State Key Laboratory of Fine Chemical Engineering, School of Life Science and Biotechnology and School of Software, Dalian University of Technology, No. 2 Linggong Road, Dalian, Liaoning 116024, People’s Republic of China; b Institute of Plant Protection, Chinese Academy of Agricultural Sciences, No. 2 West Yuanmingyuan Road, Beijing 100193, People’s Republic of China

**Keywords:** chitin, chitinase, chitin synthesis, chitin degradation, synergy

## Abstract

The glycoside hydrolase family 18 chitinases degrade or alter chitin. Multiple catalytic domains in a glycoside hydrolase family 18 chitinase function synergistically during chitin degradation. Here, an insect group III chitinase from the agricultural pest *Ostrinia furnacalis* (*Of*ChtIII) is revealed to be an arthropod-conserved chitinase that contains two nonsynergistic GH18 domains according to its catalytic properties. Both GH18 domains are active towards single-chained chitin substrates, but are inactive towards insoluble chitin substrates. The crystal structures of each unbound GH18 domain, as well as of GH18 domains complexed with hexa-*N*-acetyl-chitohexaose or penta-*N*-acetyl-chitopentaose, suggest that the two GH18 domains possess endo-specific activities. Physiological data indicated that the developmental stage-dependent gene-expression pattern of *Of*ChtIII was the same as that of the chitin synthase *Of*ChsA but significantly different from that of the chitinase *Of*ChtI, which is indispensable for cuticular chitin degradation. Additionally, immunological staining indicated that *Of*ChtIII was co-localized with *Of*ChsA. Thus, *Of*ChtIII is most likely to be involved in the chitin-synthesis pathway.

## Abstract 2

Multiple catalytic domains in one chitinase have been shown to function synergistically during chitin degradation. Here, using biochemical and structural characterization, an insect chitinase was revealed to have two nonsynergistic catalytic domains, which may be involved in chitin synthesis instead of chitin degradation.

## 1. Introduction   

The glycoside hydrolase family 18 (GH18) chitinases (EC 3.2.1.14) catalyze the breakdown of β-1,4-glycosidic bonds in chitin or chitooligosaccharides (Carbohydrate Active Enzymes database; http://www.cazy.org/; Lombard *et al.*, 2014 ▸; The CAZypedia Consortium, 2017 ▸). They are widely distributed across the tree of life and play various vital roles (Adrangi & Faramarzi, 2013 ▸). For organisms in which chitin is a structural component, such as fungi, arthropods and nematodes, chitinases are used to remodel cell walls, cuticles and eggshells, respectively (Hartl *et al.*, 2012 ▸; Zhu *et al.*, 2016 ▸). In bacteria, chitinases are produced to degrade exogenous chitin for nutrients (Vaaje-Kolstad *et al.*, 2013 ▸). In pathogenic protozoa, chitinase is used to facilitate transmission by disrupting the peritrophic matrix of insect pest vectors, such as mosquitos (Shahabuddin *et al.*, 1993 ▸). In plants, chitinases play a defensive role against microbial pathogens by targeting their cell walls and mediate plant–microorganism symbiosis by modifying signal molecules in leguminous plants (Grover, 2012 ▸). In humans, two chitinases, macrophage chitotriosidase and acidic mammalian chitinase (AMCase), have been implicated in innate immunological responses to chitin-containing pathogens (Lee *et al.*, 2011 ▸).

GH18 chitinases usually contain one catalytic GH18 domain and several auxiliary domains, such as chitin-binding domains and fibronectin type III-like domains. The crystal structures of chitinases with one single GH18 domain have been extensively studied in archaea (Tsuji *et al.*, 2010 ▸), bacteria (Perrakis *et al.*, 1994 ▸; van Aalten *et al.*, 2000 ▸; Songsiriritthigul *et al.*, 2008 ▸; Hsieh *et al.*, 2010 ▸; Busby *et al.*, 2012 ▸; Payne *et al.*, 2012 ▸; Madhuprakash *et al.*, 2013 ▸; Malecki *et al.*, 2013 ▸; Üstok *et al.*, 2015 ▸; Itoh *et al.*, 2016 ▸), fungi (Hollis *et al.*, 2000 ▸; Rao *et al.*, 2005 ▸; Hurtado-Guerrero & van Aalten, 2007 ▸; Schüttelkopf *et al.*, 2010 ▸; Yang *et al.*, 2010 ▸), plants (Terwisscha van Scheltinga *et al.*, 1994 ▸; Cavada *et al.*, 2006 ▸; Ohnuma, Numata, Osawa, Mizuhara, Lampela *et al.*, 2011 ▸; Ohnuma, Numata, Osawa, Mizuhara, Vårum *et al.*, 2011 ▸; Kitaoku *et al.*, 2015 ▸; Masuda *et al.*, 2015 ▸; Umemoto *et al.*, 2015 ▸), insects (Chen *et al.*, 2014 ▸; Liu *et al.*, 2017 ▸) and humans (Fusetti *et al.*, 2002 ▸; Olland *et al.*, 2009 ▸). Although the overall structure of these GH18 domains is a (β/α)_8_-barrel with a substrate-binding cleft on the top side, they can be differentiated by the shapes of the substrate-binding clefts and the presence or absence of insertion domains.

Chitinases with two GH18 domains have been discovered in viruses, archaea, bacteria and insects (Hiramatsu *et al.*, 1999 ▸; Tanaka *et al.*, 2001 ▸; Howard *et al.*, 2004 ▸; Arakane & Muthukrishnan, 2010 ▸; Itoh *et al.*, 2016 ▸). In some of these chitinases, the two catalytic domains work synergistically because the two domains in combination exhibit a significantly higher activity than the sum of their individual activities. In some cases, different catalytic activities of the two domains account for the synergism. In *Tk*-ChiA, a chitinase from the hyperthermophilic archaeon *Thermococcus kodakaraensis* KOD1, the N-terminal and C-terminal GH18 domains function as an exo-chitinase and an endo-chitinase, respectively (Tanaka *et al.*, 2001 ▸). This is also true for the two GH18 domains of the chitinase B from *Microbulbifer degradans* 2-40 (Howard *et al.*, 2004 ▸). A different mechanism is found for ChiW, a chitinase from *Paenibacillus* sp. FPU-7 (Itoh *et al.*, 2016 ▸). Structural studies indicate that the two GH18 domains are assembled into a compact catalytic region together with two immunoglobulin-like fold domains. This unique spatial arrangement is deduced to facilitate synergism for the efficient degradation of chitin on the cell surface.

Here, we report an arthropod-conserved insect chitinase belonging to group III from the pest *Ostrinia furnacalis* (*Of*ChtIII), which contains two GH18 domains that act without synergism according to enzymatic assays and structural investigations. *Of*ChtIII contains 987 amino-acid residues and is composed of four domains: a predicted TM domain (residues 7–29), two catalytic domains, GH18A (residues 94–461) and GH18B (residues 530–889), and a CBM14 domain (residues 922–976). Its physiological role remains elusive (Zhu *et al.*, 2008 ▸; Pesch *et al.*, 2016 ▸; Xi *et al.*, 2015 ▸; Su *et al.*, 2016 ▸). *In situ* immunological staining as well as gene-expression pattern analysis suggested that the role of *Of*ChtIII is linked to the chitin-synthesis pathway. Although chitinase is known to degrade chitin, this work suggests that some chitinases containing two nonsynergistic GH18 domains can act in chitin synthesis.

## 2. Methods   

### 2.1. Insect culture, cDNA cloning and sequence analysis of *Of*ChtIII   

*O. furnacalis* was reared as described previously (Yang *et al.*, 2008 ▸). The full-length cDNA encoding *Of*ChtIII was cloned from the total RNA of *O. furnacalis* white pupae by RT-PCR, 5′-RACE and 3′-RACE using the primers listed in Supplementary Table S1. The transmembrane (TM) segment was predicted using *TMHMM* (http://www.cbs.dtu.dk/services/TMHMM/). A *BLASTP* algorithm-based search using the amino-acid sequence of *Of*ChtIII as a query was performed, and a phylogenetic tree of 5000 sequences was generated by the web server using the default parameters (http://blast.ncbi.nlm.nih.gov/; see Supplementary Data 1).

### 2.2. Expression and purification of *Of*ChtIII, its truncations and its mutants   

DNAs encoding mature *Of*ChtIII (residues 40–987) and GH18B (residues 528–904) were amplified by PCR from the cDNA of *Of*ChtIII using the primers listed in Supplementary Table S2. The *Of*ChtIII E217L and GH18B E647L mutants were produced using the QuikChange site-directed mutagenesis kit (Stratagene, La Jolla, USA) as described by the manufacturer. The primers used are also listed in Supplementary Table S2. The resultant DNAs were cloned into the pPIC9 vector (Invitrogen, Carlsbad, USA), linearized by PmeI (Invitrogen) and transformed into *Pichia pastoris* GS115 cells. His_6_ tags were added to the C-termini of these proteins. The transformants were selected and cultured as described previously (Liu *et al.*, 2009 ▸). The cells were cultured in BMMY broth for 72 h, and methanol (1% of the total volume) was added every 24 h. The culture supernatants were then harvested by centrifugation at 8000*g* for 15 min.

The culture supernatants were concentrated by ammonium sulfate precipitation (70% saturation). The proteins were dissolved in buffer *A* (20 m*M* sodium phosphate, 0.5 *M* NaCl pH 7.4) and purified by immobilized metal ion-affinity chromatography using a HisTrap HP column (5 ml; GE Healthcare, Shanghai, People’s Republic of China). GH18 domain A (GH18A) or the GH18A E217L mutant (GH18A-E217L) was obtained in the flowthrough fraction and GH18 domain B-family 14 carbohydrate-binding module (GH18B-CBM14), GH18B and its E647 mutant (GH18B-E647L) were obtained in the elution fraction with buffer *A* containing 250 m*M* imidazole. GH18A and GH18A-E217L were further purified by cation-exchange chromatography using a RESOURCE S column (6 ml; GE Healthcare) with a linear NaCl gradient from 20 to 250 m*M*. The purified proteins were analyzed by SDS–PAGE and their concentrations were determined by measuring the absorbance at 280 nm. The purity of recombinant GH18A, GH18B-CBM14 and GH18B were confirmed by SDS–PAGE analysis.

### 2.3. N- and C-terminal sequencing by mass spectrometry   

N- and C-terminal sequencing of the proteins was carried out by ProtTech (People’s Republic of China). In brief, GH18A and GH18B-CBM14 were dissolved in 6 *M* guanidine–HCl. Cysteine residues were reduced by adding 20 m*M* DTT and were then alkylated by reaction with iodoacetamide. After desalting, the protein samples were separated by SDS–PAGE. The protein gel bands were excised and subjected to trypsin and chymotrypsin digestion. The resulting peptides from each digestion reaction were analysed by nano-ESI-LC-MS/MS. In the analysis, the peptide samples were separated by reverse-phase HPLC coupled online to an LCQ Deca XP Plus mass spectrometer (Thermo, Waltham, USA). The mass-spectrometric data were analysed using proprietary peptide-mapping software from ProtTech to map the N- and C-termini of GH18A and GH18B-CBM14.

### 2.4. Biochemical characterization of *Of*ChtIII   

The substrate specificities of GH18A and GH18B were investigated using α-chitin (Sigma–Aldrich, Shanghai, People’s Republic of China), colloidal chitin, ethylene glycol chitin (EGC; Wako, Japan) and chitooligosaccharides [(GlcNAc)_*n*_, *n* = 3–6; BZ Oligo Biotech, Qingdao, People’s Republic of China] as substrates. For the polymeric substrates, reaction mixtures contained substrate (2 mg ml^−1^) and enzyme (50 n*M*) in 200 µl of 5 m*M* sodium phosphate buffer pH 6.0. After incubation at 30°C for 1 h, the mixture was boiled for 5 min to stop the reaction. After centrifugation at 12 000*g* for 10 min, 60 µl supernatant was added to 180 µl ferri/ferrocyanide reagent (Imoto & Yagishita, 1971 ▸) and the mixture was boiled for 15 min. After centrifugation at 12 000*g* for 10 min, the supernatant was collected and the absorbance was measured at 405 nm using a Sunrise microplate reader (Tecan, Switzerland). The reaction velocity was determined by comparing the absorbance of the hydrolytic products with the standard curve for (GlcNAc)_2_ at known concentrations. To determine the kinetic parameters towards EGC, substrate concentrations from 0.066 to 0.33 mg ml^−1^ were used. The *K*_m_ and *k*_cat_ values were calculated by linear regression using Lineweaver–Burk plots.

For (GlcNAc)_*n*_, reaction mixtures contained the substrate (0.1 m*M*) and an appropriate amount of enzyme (3 n*M*) in 50 µl of 5 m*M* sodium phosphate buffer pH 6.0. After incubation at 30°C for a specific period, 10 µl of the hydrolytic products was immediately analyzed by HPLC using a TSK gel amide-80 column (4.6 × 250 mm; Tosoh, Tokyo, Japan; Koga *et al.*, 1998 ▸). The reaction velocity was calculated by comparing the reduced peak area of the substrate (GlcNAc)_*n*_ with a standard curve for (GlcNAc)_*n*_ at known concentrations.

The time-course chitin-binding assays were carried out at 25°C. A 400 µl reaction mixture, containing 80 µg protein and 4 mg of either α-chitin or β-chitin in 20 m*M* sodium phosphate buffer with 150 m*M* NaCl pH 6.0, was incubated for a time of 0, 0.5, 1, 1.5, 2, 4 or 6 h, and the supernatant was then collected by centrifugation at 12 000*g* for 5 min. The concentration of the free protein in the supernatant was determined by the Bradford method, and the concentration of the bound protein was calculated from the difference between the total protein and free protein concentration.

### 2.5. Crystallization and data collection   

Crystallization experiments were performed using the hanging-drop vapour-diffusion method at 4°C. GH18A and GH18B were desalted in 20 m*M* MES with 50 m*M* NaCl pH 6.0 and concentrated to 10 mg ml^−1^ by ultracentrifugation. GH18A and GH18B crystallized within two weeks in condition *A* (200 m*M* ammonium sulfate, 100 m*M* bis-tris pH 6.5, 20% PEG 3350) and condition *B* (200 m*M* trisodium citrate dihydrate pH 8.1, 20% PEG 3350), respectively. GH18A-E217L and GH18B-E647L crystallized within two weeks in condition *C* (200 m*M* ammonium sulfate, 100 m*M* bis-tris pH 6.6, 21% PEG 3350) and condition *B*, respectively. Crystals of the GH18A–(GlcNAc)_6_ complex were obtained by transferring native crystals to a solution consisting of 5 m*M* (GlcNAc)_6_ in condition *A*. The crystals were soaked for approximately 5 or 30 min at room temperature. Crystals of the GH18B–(GlcNAc)_3_ complex were obtained by transferring native crystals to a stabilizing solution consisting of 5 m*M* (GlcNAc)_6_ in condition *B*. The crystals were soaked for approximately 30 min at room temperature. Crystals of the GH18A-E217L–(GlcNAc)_6_ complex were obtained by transferring native crystals to a stabilizing solution consisting of 5 m*M* (GlcNAc)_6_, 200 m*M* ammonium sulfate, 100 m*M* bis-tris pH 6.6, 21% PEG 3350. The crystals were soaked for approximately 1 h at room temperature. Crystals of the GH18B-E647L–(GlcNAc)_5_ complex were obtained by transferring native crystals to a stabilizing solution consisting of 10 m*M* (GlcNAc)_5_, 200 m*M* trisodium citrate pH 8.1, 24% PEG 3350. The crystals were soaked for approximately 1 h at room temperature. These crystals were soaked for several minutes in reservoir solution containing 25% glycerol as a cryoprotecting agent and were subsequently flash-cooled in liquid nitrogen. The diffraction data were collected at Shanghai Synchrotron Radiation Facility. The diffraction data were processed using the *HKL*-2000 package (Otwinowski & Minor, 1997 ▸).

### 2.6. Structure determination and refinement   

The structures of GH18A and GH18B were determined by molecular replacement with *Phaser* (McCoy *et al.*, 2007 ▸) using the structure of human acidic mammalian chitinase (AMCase; PDB entry 3fxy; Olland *et al.*, 2009 ▸) as a model. The structures of GH18A–(GlcNAc)_6_, GH18A-E217L and GH18A-E217L–(GlcNAc)_6_ were determined by molecular replacement with *Phaser* using the structure of GH18A as a model. The structures of GH18B–(GlcNAc)_3_, GH18B-E647L and GH18B-E647L–(GlcNAc)_5_ were determined by molecular replacement with *Phaser* using the structure of GH18B as a model. *PHENIX* (Adams *et al.*, 2010 ▸) was used for structural refinement. The molecular models were manually built and extended using *Coot* (Emsley *et al.*, 2010 ▸). The stereochemistry of the models was confirmed by *PROCHECK* (Laskowski *et al.*, 1993 ▸). The atomic coordinates and structure factors have been deposited in the Protein Data Bank (http://wwpdb.org) as entries 5wup, 5wv8, 5wv9, 5wvb, 5wus, 5wvf, 5wvh and 5wvg. All structural figures were generated using *PyMOL* (Schrödinger).

### 2.7. Immunofluorescence staining   

Cuticles of one-day-old fifth-instar *O. furnacalis* were collected and fixed in 4% paraformaldehyde at 4°C overnight, dehydrated in an ascending series of ethanol concentrations, cleared in xylene and embedded in paraffin. The samples were sectioned to 5–10 µm, deparaffinized, rehydrated, heated in EDTA-based antigen-retrieval solution at 100°C for 20 min and stained for *Of*ChtIII and *O. furnacalis* chitin synthase A (*Of*ChsA) proteins using *Of*ChtIII rabbit antiserum (1:50) and *Of*ChsA guinea pig antiserum (1:10) as primary antibodies, respectively. The polyclonal antisera for *Of*ChtIII and *Of*ChsA were generated by immunizing guinea pigs with the peptide CSSFESNDETKDGKTGL and rabbits with the peptide QPRQNQVSFQRYS, respectively (GL Biochem, Shanghai, People’s Republic of China). TRITC goat anti-guinea pig IgG (1:100; ImmunoReagents, Raleigh, USA) and Alexa Fluor 488 goat anti-rabbit IgG (1:200; Jackson Immuno­Research, West Grove, USA) were used as secondary antibodies, respectively, for fluorescence detection of the proteins. Rhodamine-conjugated chitin-binding probe (0.7 mg ml^−1^) and DAPI (5 µg ml^−1^) were used for chitin and nuclei staining, respectively. Confocal microscopy was performed using an Olympus FV1000 laser scanning confocal microscope (Olympus, Tokyo, Japan) equipped with lasers capable of excitation at 405, 488 and 543 nm.

## 3. Results   

### 3.1. Sequence analysis of *Of*ChtIII   

**Figure 1:**
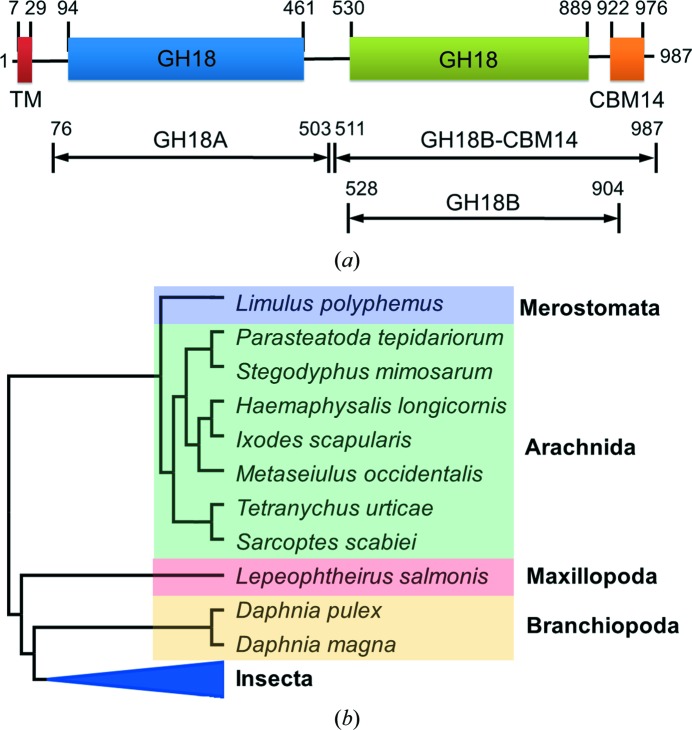
Domain and phylogenetic analysis of *Of*ChtIII. (*a*) Domain organization of *Of*ChtIII. TM, transmembrane motif; GH18, catalytic domain; CBM14, chitin-binding domain. The locations of the truncated forms of *Of*ChtIII used in this study are also shown. (*b*) Phylogenetic tree of *Of*ChtIII-like proteins from different taxa. The full phylogenetic tree, including the accession numbers of all of the protein sequences used, is provided in Supplementary Data 1.

An mRNA encoding *Of*ChtIII was cloned from *O. furnacalis* and deposited in GenBank (accession No. KF318218). *Of*ChtIII is composed of four domains: a predicted TM domain (residues 7–29), two catalytic domains, GH18A (residues 94–461) and GH18B (residues 530–889), and a CBM14 domain (residues 922–976) (Fig. 1 ▸ *a*). To understand the sequence conservation of *Of*ChtIII, a *BLASTP* search using the amino-acid sequence of *Of*ChtIII as a query was performed and a phylogenetic tree of 5000 sequences was generated (see Supplementary Data 1). The clade containing *Of*ChtIII contains sequences from insects and other arthropod classes including merostomata, arachnida, maxillopoda and branchiopoda, which range from land to ocean (Fig. 1 ▸ *b*). Moreover, the domain composition of *Of*ChtIII, GH18A-GH18B-CBM14, was conserved in this clade, with over 50% shared sequence identity. This result indicated a conserved role of *Of*ChtIII analogues in the process of chitin synthesis in the arthropod world.

### 3.2. Biochemical activity of *Of*ChtIII   

*Of*ChtIII was first produced in *P. pastoris* and its enzymatic activities towards different substrates were then determined. During expression in *P. pastoris*, the recombinanat *Of*ChtIII enzyme was found to be naturally cleaved into two active fragments: GH18A and GH18B-CBM14 (Fig. 1 ▸ *a*). The two fragments were separately purified (Supplementary Fig. S1), and the C-terminal amino-acid sequence of GH18A and the N-terminal amino-acid sequence of GH18B-CBM14 were determined by LC-MS/MS (Supplementary Figs. S2–S5), which indicated that the cleavage was between residues Arg503 and Leu511. Additionally, GH18B without the CBM14 was cloned, produced and purified in *P. pastoris* (Supplementary Fig. S1). In parallel, the enzymatic activity of the insect group I chitinase, *Of*ChtI, the physiological role of which is tightly linked to cuticle chitin degradation during moulting, was tested for comparison (Chen *et al.*, 2014 ▸).

Four forms of polymeric chitin, as well as four chito­oligosaccharides, were used as substrates for enzymatic kinetic studies. GH18A, GH18B and GH18B-CBM14 showed very similar activity patterns, with no activity towards the insoluble substrates and high activities towards the soluble substrates (Table 1 ▸). The presence of the CBM14 with GH18B did not increase its activity levels towards the insoluble substrates. For the soluble EGC substrate, the catalytic efficiencies of GH18A and GH18B were very similar (2.75 and 2.77 s^−1^ mg^−1^ ml^−1^, respectively; Supplementary Table S4). The degradation activity of GH18A and GH18B (or GH18B-CBM14) in combination towards EGC was equal to the activity calculated from the sum of the individual activities (Supplementary Fig. S6), suggesting that there was no synergistic effect between them. In contrast, *Of*ChtI showed activity towards colloidal chitin and β-chitin, but had a lower activity towards EGC than either GH18A or GH18B. For oligomeric substrates, the hydrolytic rates of GH18A and GH18B using (GlcNAc)_4_ as a substrate were one fourth of those with (GlcNAc)_6_ (Table 1 ▸). Notably, both GH18A and GH18B could not hydrolyze (GlcNAc)_3_, even after extended incubation at 30°C for 24 h. In contrast, *Of*ChtI showed the greatest hydrolytic activity for (GlcNAc)_4_ and considerable hydrolytic activity for (GlcNAc)_3_ (Table 1 ▸).

**Figure 2:**
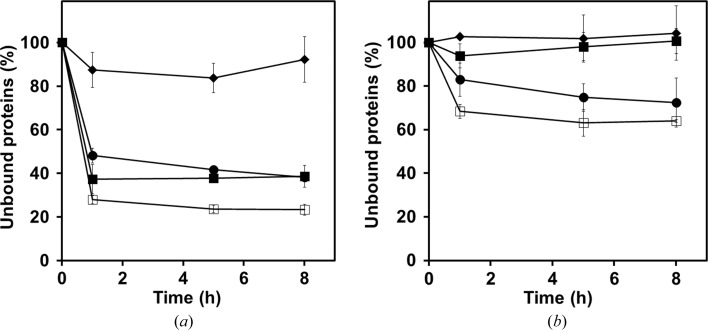
Time course for the binding of *Of*ChtIII truncates to α-chitin (*a*) and β-chitin (*b*). Filled circles, GH18A-E217L; filled squares, GH18B-E647L; open squares, GH18B-CBM14-E647L; filled diamonds, BSA.

The binding activities to α-chitin and β-chitin were determined using the active-site mutated variants GH18A-E217L, GH18B-E647L and GH18B-CBM14-E647L (Fig. 2 ▸). Both GH18A-E217L and GH18B-E647L preferentially bound α-chitin as opposed to β-chitin. GH18B-CBM14-E647L, which contained a CBM14, had much greater binding affinities for both α-chitin and β-chitin.

### 3.3. Crystal structures of GH18A and chitooligosaccharide-complexed GH18A   

GH18A crystallized in space group *P*4_1_2_1_2, and the structure was determined by molecular replacement using human AMCase as a template. In addition, the structures of two complexes were obtained: wild-type GH18A complexed with hydrolyzed (GlcNAc)_6_ and an active-site mutant, GH18A-E217L, complexed with intact (GlcNAc)_6_. The structures of the complexes were determined by molecular replacement using GH18A as a template. These structures were resolved to resolutions of between 2.0 and 3.0 Å and all data-collection and structure-refinement statistics are summarized in Table 2 ▸.

**Figure 3:**
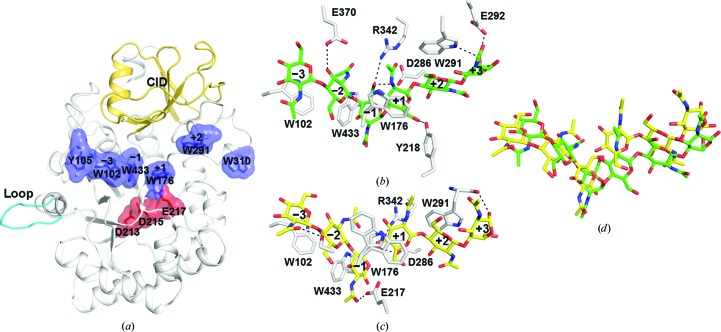
Crystal structure of GH18A and chitooligosaccharide-complexed GH18A. (*a*) A cartoon representation of the overall structure of GH18A. The TIM barrel, CID and the unique loop (residues 145–152) are shown in white, gold and cyan, respectively. The aromatic residues that line the substrate-binding groove are shown in blue and the catalytic residues are shown in red. (*b*) The intermolecular interactions between the amino-acid residues in the substrate-binding groove of GH18A-E217L and (GlcNAc)_6_. Relevant hydrogen bonds are shown as dotted lines. (*c*) The intermolecular interactions between the amino-acid residues in the substrate-binding groove of wild-type GH18A and (GlcNAc)_6_. (*d*) A structural overlay highlighting the differences between cleaved (GlcNAc)_6_ binding to wild-type GH18A (in yellow) and intact (GlcNAc)_6_ binding to GH18A-E217L (in green).

The overall structure of GH18A is a classical TIM barrel (residues 94–461) with a chitinase insertion domain (CID; residues 340–410) between strand β7 and helix α7 (Fig. 3 ▸ *a*). A unique loop (residues 145–152) is adjacent to the α7 helix. Additionally, a substrate-binding groove with lined-up aromatic residues is located on the surface. The conserved catalytic signature motif, D*X*D*X*E (residues 213–217), is in the centre of the substrate-binding groove (Fig. 3 ▸ *a*).

The structure of the GH18A-E217L–(GlcNAc)_6_ complex revealed that (GlcNAc)_6_ occupies substrate-binding groove subsites −3 to +3, where −*n* represents the reducing end and +*n* represents the nonreducing end (Davies *et al.*, 1997 ▸; Fig. 3 ▸ *b*, Supplementary Fig. S7*a*). According to Cremer–Pople parameter calculations (Hill & Reilly, 2007 ▸) and *Privateer* validation (Agirre *et al.*, 2015 ▸), most of the sugar rings are in the ^4^ *C*_1_ conformation, except for the −1 GlcNAc, which is in an unusual ^1^ *S*_5_ conformation (Table S5 and Supplementary Data 2) in which the C2 acetamido group is not positioned for catalysis. It is possible that the unusual ^1^ *S*_5_ conformation is because the GH18A-E217L mutant was used to obtain the structural complex. Many interactions are responsible for (GlcNAc)_6_ binding, most notably four stacking interactions involving the aromatic residues Trp102, Try433, Trp176 and Trp291 interacting with the −3, −1, +1, and +2 sugars, respectively, and six hydrogen bonds involving the residues Glu370, Asp286, Arg342, Tyr218, Trp291 and Glu291 interacting with the −2, −1, −1, +1, +2 and +3 sugars, respectively (Fig. 3 ▸ *b*).

The complex of wild-type GH18A with (GlcNAc)_6_ confirms the subsites and substrate-binding mode revealed by the GH18A-E217L complex. (GlcNAc)_6_ is cleaved into two (GlcNAc)_3_ molecules, which are localized in the substrate-binding groove and occupy subsites −3 to −1 and +1 to +3, respectively (Fig. 3 ▸ *c*). Like (GlcNAc)_6_ in GH18A-E217L, most sugar rings are in the ^4^ *C*_1_ conformation. After cleavage, the two (GlcNAc)_3_ molecules bind to the enzyme more weakly, allowing the non­reducing end (GlcNAc)_3_ to leave from the active-site pocket vertically, while the reducing end (GlcNAc)_3_ slips out horizontally (Fig. 3 ▸ *d*). Interestingly, the Trp176 residue at subsite +1 has different conformations before and after (GlcNAc)_6_ binding (Figs. 3 ▸ *b* and 3 ▸ *d*).

### 3.4. Crystal structures of GH18B and chitooligosaccharide-complexed GH18B   

GH18B crystallized in space group *P*4_1_2_1_2 and its structure was determined by molecular replacement using the structure of human AMCase as a template. To study the substrate-binding mode, the structures of two complexes of GH18B were crystallized and obtained: that of wild-type GH18B complexed with (GlcNAc)_3_ and that of the GH18B-E647L mutant with a bound (GlcNAc)_5_. These structures were resolved to resolutions of between 2.2 and 2.8 Å, and all data-collection and structure-refinement statistics are summarized in Table 3 ▸.

**Figure 4:**
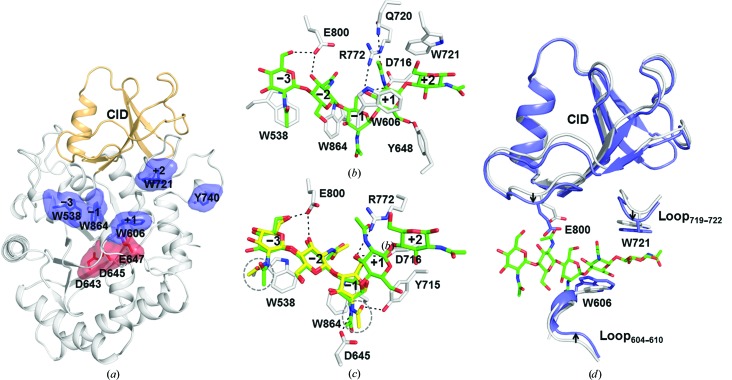
Crystal structure of GH18B and chitooligosaccharide-complexed GH18B. (*a*) Cartoon representation of the overall structure of GH18B. The TIM barrel and CID are shown in white and gold, respectively. The aromatic residues that line the substrate-binding groove are shown in blue and the catalytic residues are shown in red. (*b*) The intermolecular interactions between the amino-acid residues in the substrate-binding groove of GH18B-E647L and (GlcNAc)_5_. Hydrogen bonds are shown as black dashed lines. (*c*) The differences in the binding modes of cleaved (GlcNAc)_3_ (in yellow) in wild-type GH18B and intact (GlcNAc)_5_ (in green) in GH18A-E647L. The C2 acetamido groups with different conformations are circled. (*d*) Shrinkage of the substrate-binding groove of GH18B after (GlcNAc)_5_ binding (unliganded GH18B-E647L, white; GH18B-E647L–(GlcNAc)_5_, blue).

The overall structure of GH18B is a classical TIM barrel (residues 530–890) with a CID (residues 770–841), which is very similar to that of GH18A and has an r.m.s.d. of only 0.88 Å for 344 C^α^ atoms (Fig. 4 ▸ *a*). The substrate-binding groove on the surface is shorter than that of GH18A, perhaps because of the presence of a possible −5 subsite composed of Tyr105 in GH18A that is absent in GH18B (Fig. 4 ▸ *a*). The conserved catalytic motif D*X*D*X*E (residues 643–647) is located in the middle of the substrate-binding groove (Fig. 4 ▸ *a*).

In the structure of the GH18B-E647L–(GlcNAc)_5_ complex, (GlcNAc)_5_ is found in the substrate-binding groove and occupies five subsites from −3 to +2 (Fig. 4 ▸ *b*, Supplementary Fig. S7*b*). Most of the sugar rings are in the ^4^ *C*_1_ conformation, apart from the −1 GlcNAc, which is in the ^1^ *S*_5_ conformation (Supplementary Table S5, Supplementary Data 2). The overall conformation of (GlcNAc)_5_ in GH18B-E647L is very similar to that of (GlcNAc)_6_ in GH18A-E217L. The intermolecular interactions between GH18B-E647L and (GlcNAc)_5_ are similar to those between GH18A-E217L and (GlcNAc)_6_, but with two additional hydrogen-bonding interactions: one between C6 OH of the −3 GlcNAc and Glu800 and the other between the 2-acetamido group of the +1 GlcNAc and Gln720. In the structure of the complex of wild-type GH18B with (GlcNAc)_3_, (GlcNAc)_3_ is found to occupy three subsites from −3 to −1 (Fig. 4 ▸ *c*). The conformation of (GlcNAc)_3_ in GH18B is similar to that of (GlcNAc)_5_ in GH18B-E647L, except for the conformations of the C2 acetamido groups of the −3 and −1 GlcNAcs. The C2 acetamido group of the −1 GlcNAc is in a conformation that facilitates its O atom being positioned 3.0 Å away from the C1 atom, and the O and N atoms form hydrogen bonds to Tyr715 and Asp645, respectively.

Appreciable conformational changes are observed between the unliganded and liganded structures of GH18B (Fig. 4 ▸ *d*). The entire CID motif, the loop (residues 604–610) containing Trp606 and the loop (residues 719–722) containing Trp721 move about 1.0 Å towards the ligands, resulting in closure of the groove after ligand binding. In particular, the distance between Trp606 and Glu800 and the distance between Trp606 and Trp721 are shortened by 2.4 and 1.8 Å, respectively. In contrast, very little conformational change of GH18A was observed during ligand binding.

### 3.5. Gene-expression profile and tissue localization of *Of*ChtIII   

**Figure 5:**
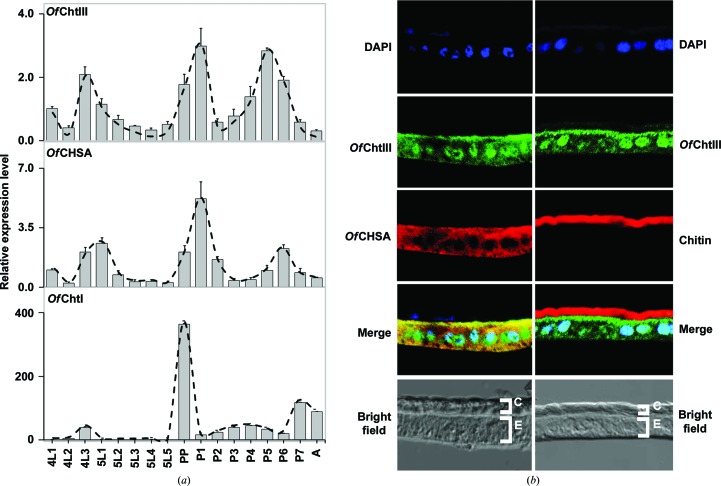
Gene-expression profile and tissue localization of *Of*ChtIII. (*a*) Expression profiles of *Of*ChtIII and related genes at different developmental stages as determined by qPCR. (*b*) Tissue localization of *Of*ChtIII and *Of*ChsA in the integument of *O. furnacalis* in one-day-old fifth instars by immunofluorescence staining. *Of*ChsA, red (left panels); chitin, red (right panels); *Of*ChtIII, green; DAPI, blue; C, cuticle; E, epidermis.

To reveal the physiological role of *Of*ChtIII, the expression profiles of *Of*ChtIII at different developmental stages were analyzed by qPCR. In addition, a representative gene in chitin synthesis, *Of*ChsA (Qu & Yang, 2011 ▸), and a representative gene in chitin degradation, *Of*ChtI (Wu *et al.*, 2013 ▸), were added as controls for comparison. The expression pattern of *Of*ChtIII was similar to that of *Of*ChsA, but differed significantly from that of *Of*ChtI (Fig. 5 ▸ *a*). The tissue localization of *Of*ChtIII in the integument of *O. furnacalis* was simultaneously determined with *Of*ChsA and chitin. *Of*ChtIII was co-localized with *Of*ChsA in the epidermal cell layer but not in the chitinous cuticle layer (Fig. 5 ▸ *b*).

## 4. Discussion   

Here, we report the first structural characterization of a chitinase containing two nonsynergistic GH18 domains. The two GH18 domains of *Of*ChtIII possess similar structures and substrate specificities, which differentiate them from the chitinolytic chitinase *Of*ChtI-CAD (Zhu *et al.*, 2008 ▸; Zhang *et al.*, 2012 ▸; Li *et al.*, 2015 ▸).

### 4.1. Structural basis for the catalytic properties of the GH18 domains of *Of*ChtIII   

The lack of synergy between GH18A and GH18B may be related to their high level of similarity. Firstly, the sequence identity of 56% between the two GH18 domains of *Of*ChtIII is much higher than those between synergistic GH18 domains (17% for chitinase A and 25% for chitinase B). Secondly, the structures of GH18A and GH18B are very similar, with an r.m.s.d. of 0.88 Å for 344 C^α^ atoms.

**Figure 6:**
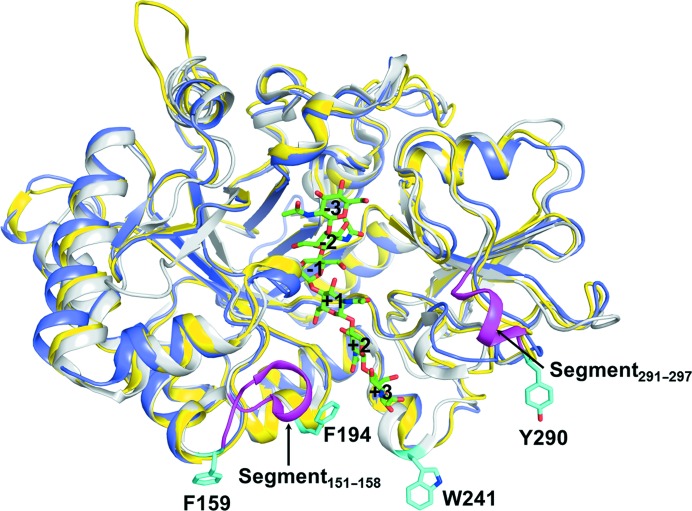
Structural comparison of GH18A (yellow), GH18B (blue) and *Of*ChtI-CAD (white). The additional segments responsible for increasing the depth of the substrate-binding cleft and the residues comprising the chitin-binding plane in *Of*ChtI-CAD are shown in pink and cyan, respectively. The (GlcNAc)_6_ in the structure of the complex of GH18A-E217L with (GlcNAc)_6_ is shown in green.

Both GH18 domains have uncommon substrate specificities, with a preference for single chitin chains (EGC) but no activity towards insoluble chitin substrates (colloidal chitin, β-chitin and α-chitin) (Table 1 ▸). To determine why *Of*ChtIII has such a substrate specificity, a structural comparison of *Of*ChtIII and *Of*ChtI was performed. Although the overall structures of GH18A and GH18B are similar to that of *Of*ChtI-CAD, with r.m.s.d.s of 1.3 Å (for 367 C^α^ atoms) and 1.5 Å (for 360 C^α^ atoms), respectively, there are obvious differences between the two enzymes. Firstly, GH18A and GH18B from *Of*ChtIII do not have the same surface hydrophobic planes as found in the GH18 domain of *Of*ChtI (characterized by Phe159, Phe194, Trp241 and Tyr290; Fig. 6 ▸). The plane in *Of*ChtI-CAD is important for the binding and hydrolysis of α-chitin (Chen *et al.*, 2014 ▸). Secondly, both GH18 domains have shorter and shallower substrate-binding clefts than *Of*ChtI-CAD. As calculated by the *CASTp* software with default parameters, the volumes of the substrate-binding clefts of *Of*ChtI-CAD, GH18A and GH18B were estimated to be 1628, 1399 and 1100 Å^3^, respectively (Dundas *et al.*, 2006 ▸). This may be partially because they do not contain the two structural segments responsible for increasing the depth of the substrate-binding cleft in *Of*ChtI-CAD (residues 151–158 and 291–297; Fig. 6 ▸).

### 4.2. A deduced role for *Of*ChtIII   

Multiple catalytic domains within one chitinase efficiently degrade chitin through synergistic actions (Tanaka *et al.*, 2001 ▸; Howard *et al.*, 2004 ▸). However, the two nonsynergistic GH18 domains in *Of*ChtIII suggest that chitinase may play a role other than in chitin degradation. This hypothesis is supported by the fact that both GH18 domains are enzymatically inactive towards insoluble chitin substrates (such as α-chitin), which is the major form of chitin in insect cuticles. The question is why are these GH18 domains highly active towards soluble chitin substrates [EGC and (GlcNAc)_*n*_], which contain single chitin chains? Why is there no need for synergism?

*Of*ChtIII contains a unique domain composition TM-GH18A-GH18B-CBM14 (Fig. 1 ▸). The presence of a TM domain is in agreement with the co-localization of the chitin synthase *Of*ChsA, which is a membrane-integrated enzyme. The orthologue *Bm*ChtIII from *Bombyx mori* (silkworm), which does not appear in either the larval cuticle (Dong *et al.*, 2016 ▸) or moulting fluid (Qu *et al.*, 2014 ▸), may provide indirect evidence for its location, although the orthologue *Tc*Cht7 from *Tribolium castaneum* (red flour beetle) is observed in the newly formed procuticle of the elytra (Noh & Arakane, 2013 ▸). This specific cellular co-localization, together with the same gene-expression patterns as those of *Of*ChsA, suggests a role in the chitin-synthesis pathway.

Carbohydrate-binding modules (CBMs) are ubiquitous domains that are able to bind polysaccharides (Boraston *et al.*, 2004 ▸). On the basis of amino-acid sequence similarity, CBMs have been divided into 83 families (Lombard *et al.*, 2014 ▸). The members of family 14 (CBM14s) are short modules that bind explicitly to chitin (Chang & Stergiopoulos, 2015 ▸). The C-terminal CBM14 improves the binding affinity of GH18B to chitin (Fig. 2 ▸), but it does not affect the hydrolytic activity of GH18B towards insoluble chitin substrates (Table 1 ▸). Therefore, the role of the CBM14 is probably to anchor *Of*ChtIII to an insoluble chitin plane, raising the question of how a chitinase could anchor to an insoluble chitin plane but act with a single-chained chitin substrate. Chitin synthesis meets these criteria. The oligomerization of chitin synthase is crucial for the generation of insoluble chitin fibrils, as pre-aligned catalytic units could facilitate the proper alignment of nascent sugar chains before coalescence (Sacristan *et al.*, 2013 ▸; Gohlke *et al.*, 2017 ▸). Evidence of trimeric ChsB complexes from the larval midgut of *Manduca sexta* (tobacco hornworm) have been reported (Maue *et al.*, 2009 ▸), which are presumed to be further oligomerized to form higher-order complexes. Thus, we hypothesized that *Of*ChtIII is localized between newly synthesized chitin chains produced by an oligomerized chitin synthase complex and a newly formed insoluble chitin fibril. The role of the C-terminal CBM14 is to facilitate the anchoring of the two active catalytic domains of *Of*ChtIII to the chitin fibril.

**Figure 7:**
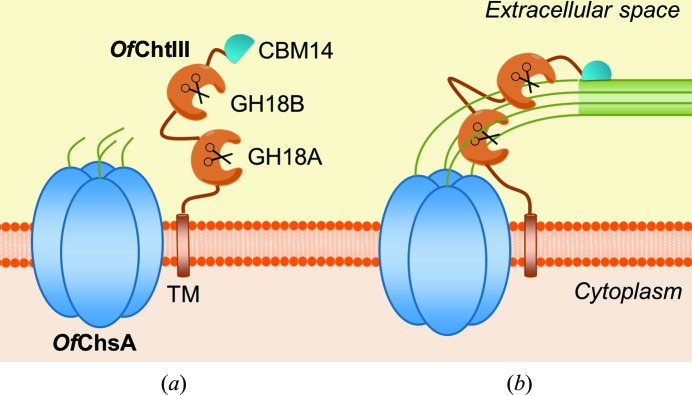
Model illustrating the physiological role of *Of*ChtIII. (*a*) The standby state; (*b*) the hydrolysis state.

Based on the above analysis, we created a model for the hypothesized role of *Of*ChtIII. At the beginning, *Of*ChtIII is in a standby state waiting for the formation of a chitin fibril (Fig. 7 ▸ *a*). Once the fibril has been formed, the CBM14 of *Of*ChtIII is anchored and the two GH18 domains are able to approach the nascent single chains (Fig. 7 ▸ *b*). There is no need for synergism because the physiological substrate of *Of*ChtIII is in the single-chained form, which is highly accessible to endochitinases.

## Supplementary Material

PDB reference: GH18A, 5wup

PDB reference: complex with (GlcNAc)_6_, 5wv9

PDB reference: E217L mutant, 5wv8

PDB reference: E217L mutant, complex with (GlcNAc)_6_, 5wvb

PDB reference: GH18B, 5wus

PDB reference: complex with (GlcNAc)_3_, 5wvh

PDB reference: E647L mutant, 5wvf

PDB reference: E647L mutant, complex with (GlcNAc)_5_, 5wvg

Supplementary Figures and Tables.. DOI: 10.1107/S2059798317018289/jt5022sup1.pdf

Supplementary Data 1. Phylogenetic tree data.. DOI: 10.1107/S2059798317018289/jt5022sup2.txt

Supplementary Data 2. Privateer validation reports.. DOI: 10.1107/S2059798317018289/jt5022sup3.pdf

## Figures and Tables

**Table 1:** Specific activity of truncated *Of*ChtIII towards different substrates ND denotes that no hydrolytic products were detected during the assay. A dash denotes not determined.

		Specific activity (µmol min^−1^ per µmol of enzyme)			
	Substrate	GH18A	GH18B	GH18B-CBM14	*Of*ChtI
Polymeric substrates†	α-Chitin	ND	ND	ND	(2.4 ± 0.4)‡
	β-Chitin	ND	ND	ND	14.9 ± 1.8
	Colloidal chitin	ND	ND	ND	37.9 ± 1.5
	EGC	222.4 ± 4.5	257.6 ± 7.8	223.7 ± 10.4	45.7 ± 5.1
Oligomeric substrates§	(GlcNAc)_6_	1434 ± 83	1381 ± 74	—	1155 ± 33
	(GlcNAc)_5_	1373 ± 13	1060 ± 56	—	1708 ± 51
	(GlcNAc)_4_	352 ± 19	340 ± 26	—	2470 ± 74
	(GlcNAc)_3_	ND	ND	—	650 ± 26

† Specific activities were determined by the reducing-sugar assay.

‡ The data in parentheses were obtained using 500 n*M* *Of*ChtI. When 50 n*M* *Of*ChtI was used, very few hydrolytic products were produced.

§ Specific activities were determined by HPLC.

**Table 2:** Details of data collection and structure refinement for GH18A-related crystals Values in parentheses are for the outer shell.

	GH18A	GH18A-E217L	GH18A–(GlcNAc)_6_	GH18A-E217L–(GlcNAc)_6_
Data collection				
Space group	*P*4_1_2_1_2	*P*4_1_2_1_2	*P*4_1_2_1_2	*P*4_1_2_1_2
Wavelength (Å)	0.97915	0.97903	0.97930	0.97850
*a*, *b*, *c* (Å)	71.835, 71.835, 193.290	72.165, 72.165, 193.031	71.781, 71.781, 193.435	72.165, 72.165, 193.031
α, β, γ (°)	90.0, 90.0, 90.0	90.0, 90.0, 90.0	90.0, 90.0, 90.0	90.0, 90.0, 90.0
Resolution (Å)	44.96–2.30 (2.42–2.30)	50–2.03 (2.07–2.03)	50–2.10 (2.14–2.10)	50–3.10 (3.15–3.10)
*R*_sym_ or *R*_merge_	0.109 (0.248)	0.076 (0.371)	0.095 (0.487)	0.115 (0.444)
〈*I*/σ(*I*)〉	17.18 (9.45)	10.6 (7.65)	9.0 (6.02)	5.7 (4.04)
Completeness (%)	99.94 (100)	94.4 (85.0)	99.9 (100)	99.9 (100)
Multiplicity	10.6 (12.1)	14.1 (12.7)	14.1 (14.4)	9.9 (9.3)
Refinement				
Resolution (Å)	2.3	2.04	2.1	3.1
No. of reflections				
Total	248482	1201224	1692705	1866296
Unique	23352	35271	30323	9906
*R*_work_/*R*_free_	0.188/0.217	0.165/0.190	0.168/0.200	0.183/0.225
No. of atoms				
Total	3533	3482	3525	3202
Protein	3123	3123	3137	3115
Ligand/ion	0	0	86	85
Water	410	359	302	2
*B* factors (Å^2^)	19.27	26.25	27.55	61.79
Protein	18.66	25.32	26.37	61.67
Ligand			36.39	66.22
Water	23.99	34.35	37.27	58.13
Root-mean-square deviations				
Bond lengths (Å)	0.008	0.007	0.010	0.09
Bond angles (°)	1.109	0.987	1.368	1.168
Ramachandran plot				
Most favoured (%)	91.7	92.2	92.6	89.6
Additionally allowed (%)	8.3	7.8	7.4	10.4
PDB code	5wup	5wv8	5wv9	5wvb

**Table 3:** Details of data collection and structure refinement for GH18B-related crystals Values in parentheses are for the outer shell.

	GH18B	GH18B-E647L	GH18B–(GlcNAc)_3_	GH18B-E647L–(GlcNAc)_5_
Data collection				
Space group	*P*4_1_2_1_2	*P*4_1_2_1_2	*P*4_1_2_1_2	*P*4_1_2_1_2
Wavelength (Å)	0.97856	0.97869	0.97945	0.97901
*a*, *b*, *c* (Å)	71.889, 71.889, 177.38	71.475, 71.475, 175.985	71.011, 71.011, 173.377	71.362, 71.362, 175.023
α, β, γ (°)	90.0, 90.0, 90.0	90.0, 90.0, 90.0	90.0, 90.0, 90.0	90.0, 90.0, 90.0
Resolution (Å)	50–2.20 (2.24–2.20)	30–2.40 (2.44–2.40)	50–2.80 (2.85–2.80)	30–2.69 (2.79–2.69)
*R*_sym_ or *R*_merge_	0.078 (0.375)	0.067 (0.381)	0.094 (0.499)	0.126 (0.467)
〈*I*/σ(*I*)〉	13.6 (7.26)	11.1 (6.61)	7.1 (6.02)	7.5 (5.19)
Completeness (%)	98 (100)	99.6 (100)	100 (100)	100 (100)
Multiplicity	14.1 (14.9)	13.5 (13.9)	13.4 (13.7)	13.6 (13.9)
Refinement				
Resolution (Å)	2.2	2.4	2.8	2.69
No. of reflections				
Total	126844	1500303	1265752	1182073
Unique	24519	18731	11615	13275
*R*_work_/*R*_free_	0.190/0.219	0.207/0.233	0.211/0.244	0.205/0.237
No. of atoms				
Total	3189	3119	3036	3090
Protein	3005	3004	2925	2964
Ligand/ion		14	71	85
Water	184	101		41
*B* factors (Å^2^)	40.25	44.10	48.22	44.43
Protein	39.72	43.81	47.80	44.38
Ligand		69.26	65.82	44.90
Water	48.89	49.21		47.46
Root-mean-square deviations				
Bond lengths (Å)	0.013	0.008	0.008	0.011
Bond angles (°)	1.38	1.13	1.28	1.22
Ramachandran plot				
Most favoured (%)	89.6	90.5	90.1	91.6
Additionally allowed (%)	10.4	9.5	9.9	8.4
PDB code	5wus	5wvf	5wvh	5wvg
