# Supplementary material for: The deduced role of a chitinase containing two nonsynergistic catalytic domains
Source: Acta Crystallogr D Struct Biol. 2018 Jan 1;74(Pt 1):30–40. doi: 10.1107/S2059798317018289 (PMC5786006; doi:10.1107/S2059798317018289)
Supplement: Supplementary file 1 [file d-74-00030-sup1.pdf]

Acta Cryst D STRUCTURAL BIOLOGY

Volume 74 (2018)

Supporting information for article:

The deduced role of a chitinase containing two nonsynergistic catalytic domains

Tian Liu, Weixing Zhu, Jing Wang, Yong Zhou, Yanwei Duan, Mingbo Qu and Qing Yang

Table S1 The strategy and primers for the cloning of the gene encoding *Of*ChtIII.

PCR fragment	Size (bp)	Primer	Primer sequence (5′ - 3′)
OfCHTIII-a	194	*Of*ChtIII-F1	TCAGCTAGCGTGAACAGACCAAA
		*Of*ChtIII-R1	TCAAAGTTCCTGTCCCTCAAGTA
		*Of*ChtIII-F2	ATTCAGCCCGATCTCTGCACCCA
		*Of*ChtIII-R2	AACTTCTGAGTCCCGAAAGACCA
OfCHTIII-b	760	3′ RACE outer primer	TACCGTCGTTCCACTAGTGATTT
		*Of*ChtIII-F3	TGCACCCACATCATCTTCGCCTTC
		3′ RACE inner primer	CGCGGATCCTCCACTAGTGATTTCACTATAGG
		*Of*ChtIII-F4	ATGAGACCAAGGATGGCAAGACCG
OfCHTIII-c	2759	3′ RACE outer primer	TACCGTCGTTCCACTAGTGATTT
		*Of*ChtIII-F5	AGGTGTGTGAGATTCTTCGCAACG
		3′ RACE inner primer	CGCGGATCCTCCACTAGTGATTTCACTATAGG
		*Of*ChtIII-F6	GACGACGAAATGAAGGTGCCGTAC
OfCHTIII-d	772	5′ RACE outer primer	GCTGATGGCGATGAATGAACACTG
		*Of*ChtIII-R3	GGTAGGGGATGGCTGAGTAGATGA
		5′ RACE inner primer	CGCGGATCCGAATTAATACGACTCACTATAGG
		*Of*ChtIII-R4	GGGTCGCTGACATCTCCTTGAACT

Table S2 Sequences of primers for cloning of *Of*ChtIII and its mutants and truncations.

	Primer	Primer sequence (5′ - 3′)
*Of*ChtIII	*Of*ChtIII-F	GAGAGGCTGAAGCTTACGTAGAATTCGTCTCCGTCACATCCTCGGTGTCC
	*Of*ChtIII-R	AATTCGCGGCCGCCTAATGATGATGATGATGATGAGCGGCCGGTGCTTGC
CAD2	CAD2-F	AGCTTACGTAGAATTCGAGCCCCAAGTCCTCTGCTAC
	CAD2-R	AATTAATTCGCGGCCGCCTAATGATGATGATGATGATGTGACTCGTAAGGTCCGTCG
CAD1-E217L	E217L-F	TGGACATCGATTGGTTGTACCCTAAGGGCGGAGAC
	E217L-R	CCTTAGGGTACAACCAATCGATGTCCAGACCGTCG
CAD2-E647L	E647L-F	TGGACGTCGACTGGTTGTACCCAAGAGGAGCAGAT
	E647L-R	CTCTTGGGTACAACCAGTCGACGTCCAGACCGTTG

Table S3 Sequences of primers for quantitative RT-PCR.

Gene name	Primer name	Primer sequence (5′ - 3′)
OfCHTI	*OfCHTI*-F	GGCGACCCTATTCCTACCACGAC
	OfCHTI-R	GCGCCTCTTCCTCCGTCGTC
OfCHTIII	*OfCHTIII*-F	AGCCCGAACTCTGCACCC
	OfCHTIII-R	AACCCAGTCTTGCCATCCTTG
OfCHSA	*OfCHSA*-F	ACGGATTGGATGATGATTACGAC
	OfCHSA-R	CGTCCAAAGTGCCAATGTTCC
RPS3	*OfRPS3*-F	TGCAACGACTACGTCAACACC
	OfRPS3-R	TCGGGCTGCGGTTTCTT

Table S4 Kinetic parameters of CAD1 and CAD2 toward ethyl glycol chitin.

	K_m (mg ml^{-1})	k_{cat} (s^{-1})	k_{cat}/K_m (s^{-1} mg^{-1} ml)
CAD1	1.10±0.09	3.02±0.19	2.75
CAD2	2.19±0.17	6.06±0.11	2.77

Table S5. The results of Cremer-Pople parameter calculation of −1 GlcNAcs.

	φ (°)	θ (°)
Pyranose in ideal $^{1}S_5$ conformation	270	90
−1 GlcNAc of $(GlcNAc)_6$ in GH18A-E217L	262	90
−1 GlcNAc of $(GlcNAc)_5$ in GH18B-E647L	257	88

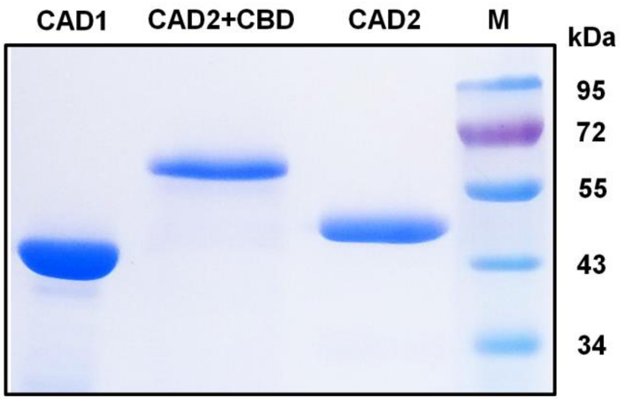

Figure S1 SDS-PAGE analysis of the purified CAD1, CAD2-CBD and CAD2.

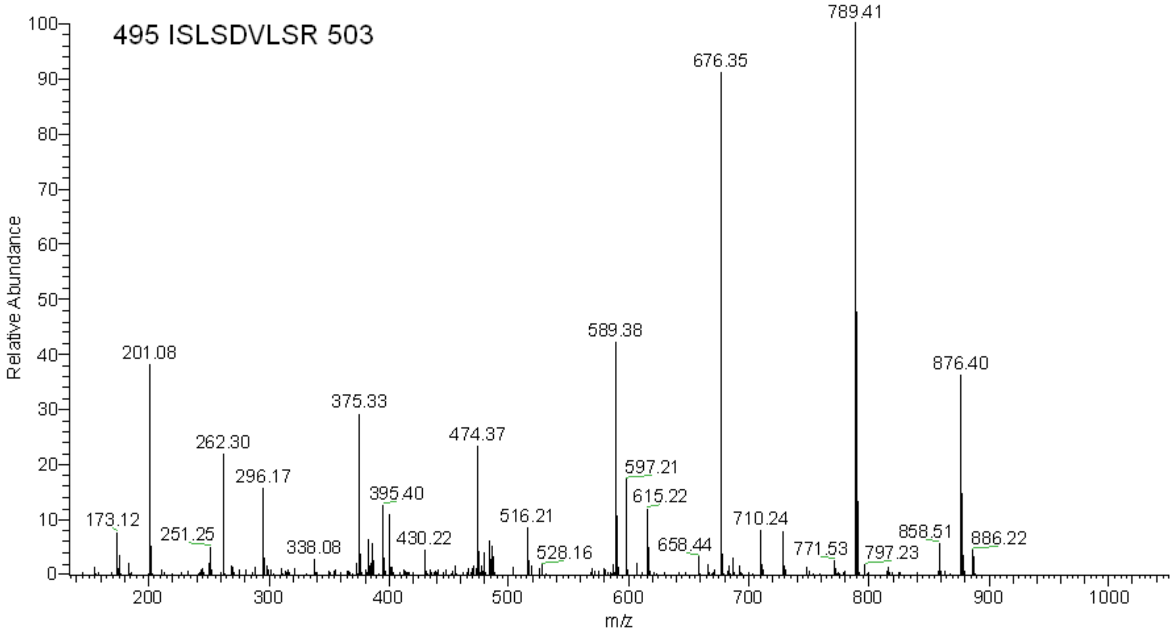

Figure S2 MS/MS spectrum of C-terminal peptide of CAD1 from trypsin digestion.

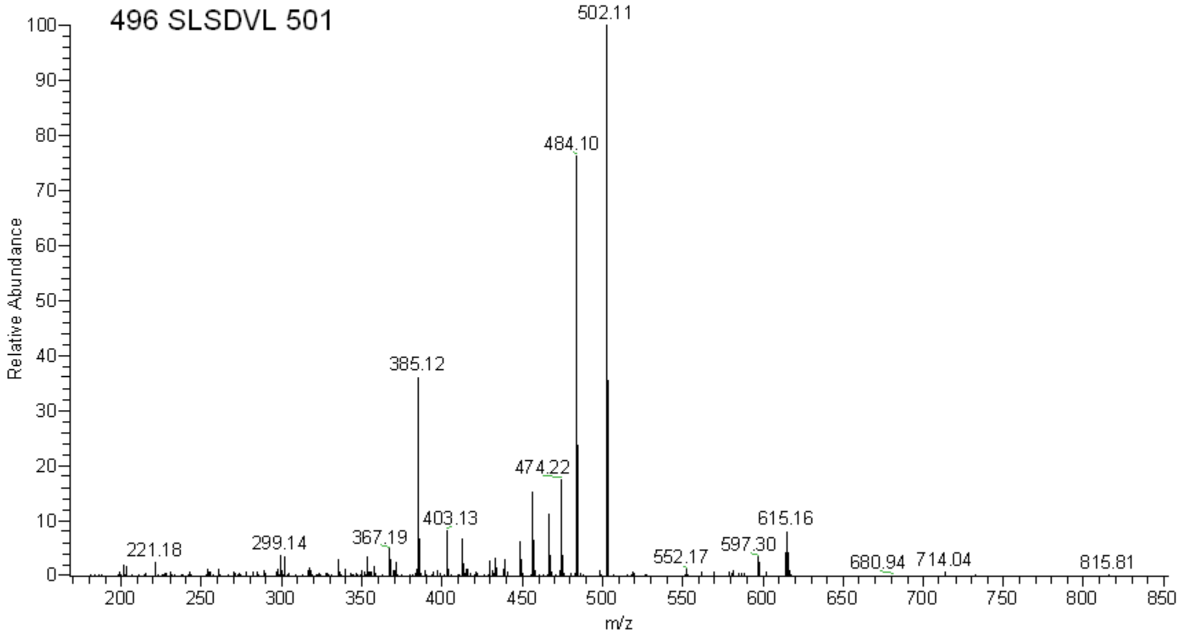

Figure S3 MS/MS spectrum of C-terminal peptide of CAD1 from chymotrypsin digestion.

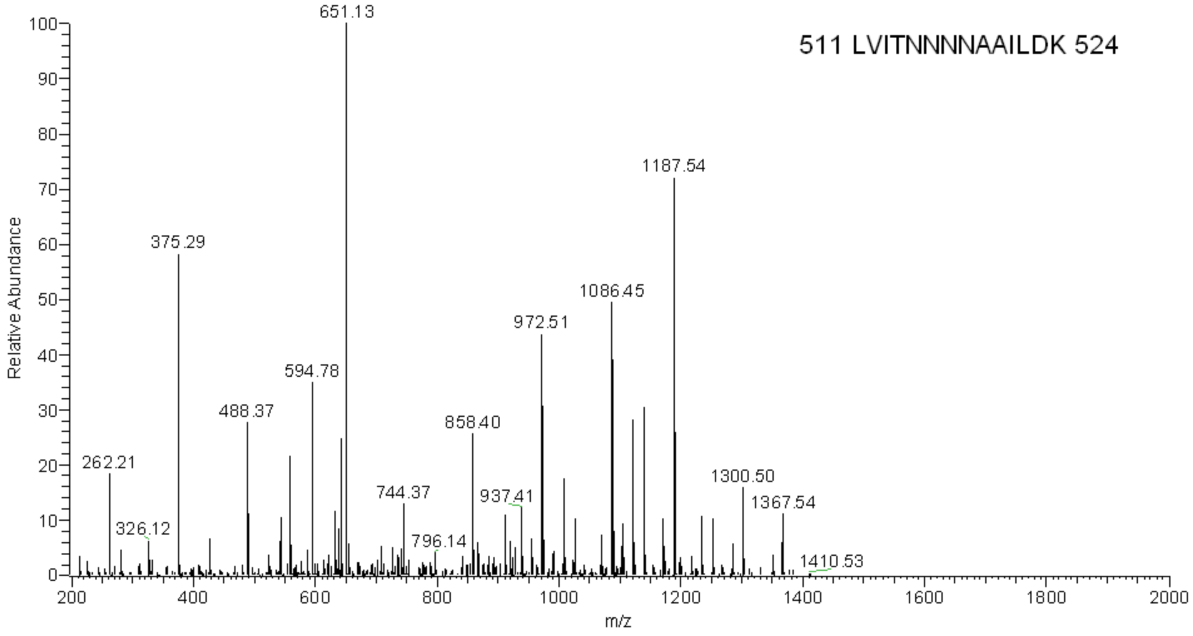

Figure S4 MS/MS spectrum of N-terminal peptide of CAD2 from trypsin digestion.

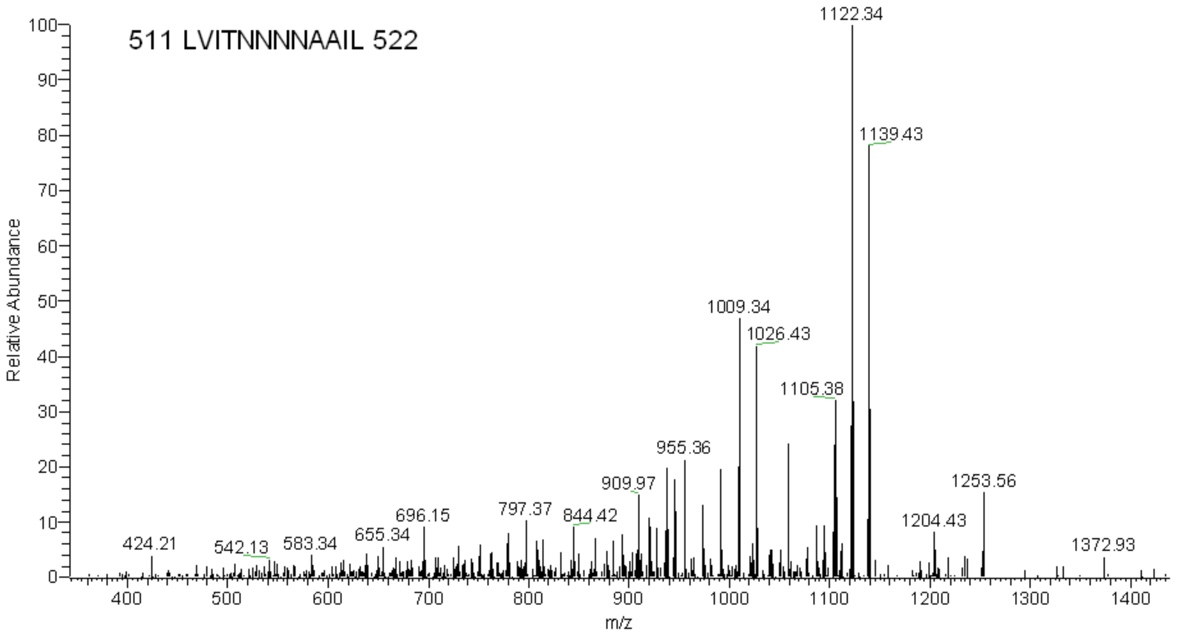

Figure S5 MS/MS spectrum of N-terminal peptide of CAD2 from chymotrypsin digestion.

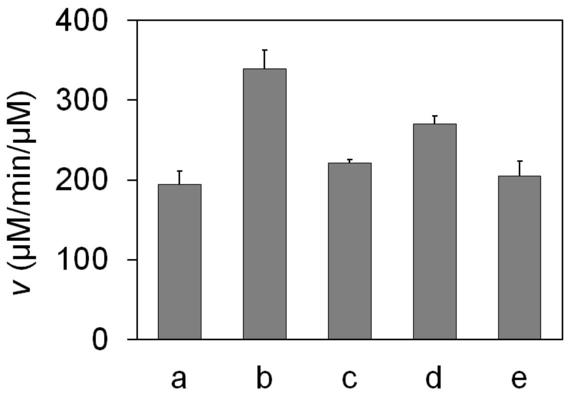

Figure S6 Synergism of GH18A, GH18B and GH18B-CBM14 on the degradation of ethylene glycol chitin. a, GH18A; b, GH18B; c, GH18B-CBM14; d, GH18A+GH18B; e, GH18A, GH18B-CBM14.

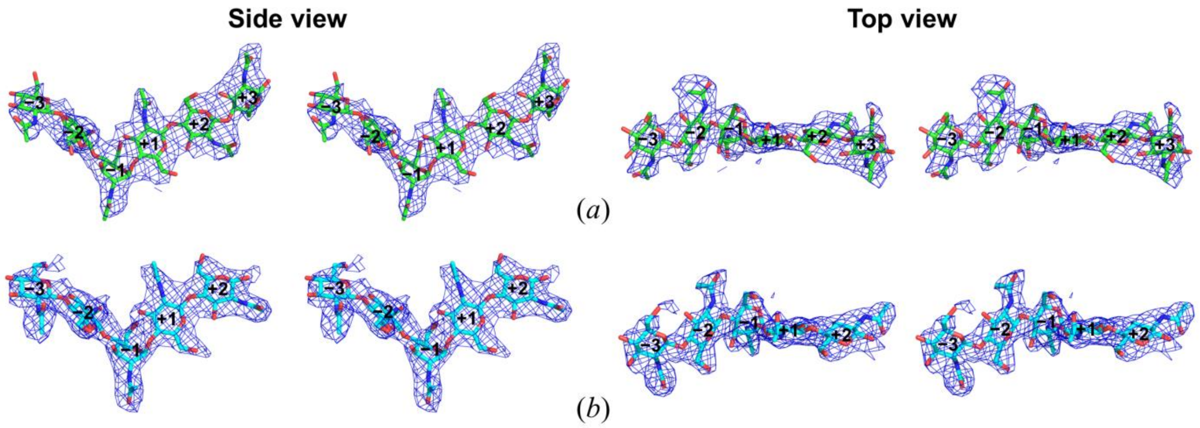

Figure S7 The stereo views of the simulated-annealing composite omit maps of $(GlcNAc)_6$ in GH18A-E217L (*a*) and $(GlcNAc)_5$ in GH18B-E647L (*b*). The composite omit map was calculated using *PHENIX* program with simulated-annealing at 3,000 K. The $2F_o-F_c$ map around the ligand is contoured at the 1.0 σ level.
