# Supplementary material for: The deduced role of a chitinase containing two nonsynergistic catalytic domains
Source: Acta Crystallogr D Struct Biol. 2018 Jan 1;74(Pt 1):30–40. doi: 10.1107/S2059798317018289 (PMC5786006; doi:10.1107/S2059798317018289)
Supplement: Supplementary file 3 [file d-74-00030-sup3.pdf]

STRUCTURAL BIOLOGY

Volume 74 (2018)

Supporting information for article:

The deduced role of a chitinase containing two nonsynergistic catalytic domains

Tian Liu, Weixing Zhu, Jing Wang, Yong Zhou, Yanwei Duan, Mingbo Qu and Qing Yang

Validation result by Privateer: GH18A-E217L-(GlcNAc)$_6$

```
<B><FONT COLOR='#FF0000'><!--SUMMARY_BEGIN-->
<html> <!-- CCP4 HTML LOGFILE -->
<hr>
<pre>

 ###############################################################
 ###############################################################
 ###############################################################
 ### CCP4 6.5: privateer-validate     version MKII : 06/01/15##
 ###############################################################
 User: yzhou  Run date: 29/11/2017 Run time: 15:46:44


 Please reference: Collaborative Computational Project, Number 4. 2011.
 "Overview of the CCP4 suite and current developments". Acta Cryst. D67, 235-242.
 as well as any specific reference in the program write-up.

<!--SUMMARY_END--></FONT></B>
```

Copyright 2013-2015 Jon Agirre, Kevin Cowtan and The University of York.

```
pdbin      ofChtIII_refine_4.pdb

Reading ofChtIII_refine_4.pdb... done.

Analysing carbohydrates...

Number of detected glycans: 0

Detailed validation data
------------------------
```

PDB	Sugar	Q	Phi	Theta	Detected type	Cnf	<Bfac>	Bonds	Angles	Ctx	Ok?
ofCh	NAG-L-1	0.550	102.27	14.38	beta-D-aldopyranose	4c1	77.73	0.015	2.139	(1)	yes
ofCh	NAG-L-2	0.571	312.51	4.37	beta-D-aldopyranose	4c1	69.00	0.006	1.936	(1)	yes
ofCh	NAG-L-3	0.558	343.54	8.23	beta-D-aldopyranose	4c1	56.06	0.008	2.365	(1)	yes
ofCh	NAG-L-4	0.713	261.67	90.14	beta-D-aldopyranose	1s5	58.32	0.009	3.395	(1)	check
ofCh	NAG-L-5	0.577	327.55	4.26	beta-D-aldopyranose	4c1	59.28	0.006	1.719	(1)	yes
ofCh	NAG-L-6	0.598	327.77	4.02	beta-D-aldopyranose	4c1	76.09	0.007	1.498	(1)	yes

```
The results for those monosaccharides marked with (*) correspond to the first of at least two possible
conformations, each with occupancy < 1.0

SUMMARY:

   Check ring geometry: 0
   Wrong anomer: 0
   Wrong configuration: 0
   Unphysical puckering amplitude: 0
   In higher-energy conformations: 1

   Privateer-validate has identified 1 issues, with 1 of 6 sugars affected.

<B><FONT COLOR='#FF0000'><!--SUMMARY_BEGIN-->
privateer-validate: Normal termination
Times: User:      0.1s System:    0.0s Elapsed:    0:00
</pre>
```

```
</html>
<!--SUMMARY_END--></FONT></B>
```

Validation result by Privateer: GH18B-E647L-(GlcNAc)$_5$

```
<B><FONT COLOR='#FF0000'><!--SUMMARY_BEGIN-->
<html> <!-- CCP4 HTML LOGFILE -->
<hr>
<pre>

 ###############################################################
 ###############################################################
 ###############################################################
 ### CCP4 6.5: privateer-validate     version MKII : 06/01/15##
 ###############################################################
 User: yzhou  Run date: 29/11/2017 Run time: 15:46:57


 Please reference: Collaborative Computational Project, Number 4. 2011.
 "Overview of the CCP4 suite and current developments". Acta Cryst. D67, 235-242.
 as well as any specific reference in the program write-up.

<!--SUMMARY_END--></FONT></B>

Copyright 2013-2015 Jon Agirre, Kevin Cowtan and The University of York.

pdbin      ofChtIII_refine_8.pdb

Reading ofChtIII_refine_8.pdb... done.

Analysing carbohydrates...

Number of detected glycans: 1

Chain L
-------

GlcNac1600-b-ASN778


Detailed validation data
------------------------

PDB      Sugar          Q    Phi       Theta       Detected type        Cnf   <Bfac>     Bonds Angles     Ctx
      Ok?
---- ------------    ----- ------     ------     -------------------  ---   ------     ----- ------     ---
     -----
ofCh  NAG-L-1498 0.576 325.26     3.16  beta-N-aldopyranose   4c1   51.74 0.004 1.597 (l)   yes
ofCh  NAG-L-1499 0.588 278.20     4.67  beta-N-aldopyranose   4c1   27.80 0.007 1.641 (l)   yes
ofCh  NAG-L-1500 0.739 257.11     87.70 beta-N-aldopyranose   1s5   22.68 0.012 2.927 (l)   check
ofCh  NAG-L-1501 0.538 359.92     8.06  beta-N-aldopyranose   4c1   21.79 0.005 2.323 (l)   yes
ofCh  NAG-L-1502 0.568 349.47     10.43 beta-N-aldopyranose   4c1   29.66 0.005 2.159 (l)   yes
ofCh  NAG-L-1600 0.565 282.79     9.70  beta-D-aldopyranose   4c1   70.66 0.006 2.922 (n)   yes

The results for those monosaccharides marked with (*) correspond to the first of at least two possible
conformations, each with occupancy < 1.0

SUMMARY:

   Check ring geometry: 0
   Wrong anomer: 0
   Wrong configuration: 0
   Unphysical puckering amplitude: 0
   In higher-energy conformations: 1
```

```
   Privateer-validate has identified 1 issues, with 1 of 6 sugars affected.

<B><FONT COLOR='#FF0000'><!--SUMMARY_BEGIN-->
privateer-validate: Normal termination
Times: User:      0.1s System:   0.0s Elapsed:    0:00
</pre>
</html>
<!--SUMMARY_END--></FONT></B>
```
